# COL11A1 is Downregulated by miR-339-5p and Promotes Colon Carcinoma Progression

**DOI:** 10.1155/2022/8116990

**Published:** 2022-05-28

**Authors:** Weizhi Liu, Ke Meng

**Affiliations:** Department of Anorectal Clinic, The Third Affiliated Hospital of Liaoning University of Traditional Chinese Medicine, Shenyang, Liaoning 110003, China

## Abstract

The roles of COL11A1 in cancer have been increasingly considered, but the understandings of the effects of COL11A1 on colon carcinoma progress are much limited yet. qRT-PCR and Western blot were utilized to evaluate COL11A1 expression at mRNA and protein levels, respectively, in colon carcinoma cell lines. Afterward, the tumorigenesis biological effects of COL11A1 were examined by CCK-8, colony formation, Transwell, and wound healing methods. Moreover, upstream miRNAs containing the binding sites with COL11A1 were predicted by the bioinformatics methods. The interplay between COL11A1 and miR-339-5p was identified by a dual-luciferase assay. COL11A1 expression was prominently upregulated in colon carcinoma cell lines relative to that in normal human colon mucosal epithelial cell lines, and it was related to tumor stages. The outcomes of *in-vitro* experiments suggested that interfering with COL11A1 remarkably repressed the malignant behaviors of SW480 and SW620 cells. MiR-339-5p was markedly lowly expressed in colon carcinoma cell lines. Furthermore, miR-339-5p directly targeted and negatively regulated COL11A1 expression. COL11A1 upregulation promoted colon carcinoma cell functions, while overexpressing miR-339-5p evidently attenuated the promotion. These results proved the modulation of the miR-339-5p/COL11A1 axis in colon carcinoma cells, and miR-339-5p repressed colon carcinoma progression via COL11A1 downregulation. These results offer new underlying targets for the accurate therapy of colon carcinoma patients.

## 1. Introduction

Colon carcinoma is the third major cause of cancer-associated deaths (more than 50,000 deaths each year) [1]. Most colon carcinoma patients have regional or distant metastases at the time of diagnosis, and therefore adjuvant chemotherapy or palliative chemotherapy for metastatic disease after surgery is required [2]. For patients with metastasis, treatment strategies include chemotherapy drugs, and biologics and immunotherapy that target vascular endothelial growth factor and epidermal growth factor receptor pathways. Two phase 3 trials investigated a prolonged survival in the third-line setting of colorectal cancer patients who received regorafenib (a tyrosine kinase inhibitor) with the longest treatment duration of 16 months [3]. Derakhshani's study discovered that capecitabine, as a prodrug of 5-FU, significantly inhibits CTLA-4 expression in SW480 cells, which may associate immunotherapy with chemotherapy [4]. The overall morbidity of colon carcinoma has been falling in the past few decades, and the overall 5-year survival rate has increased to 64% so far due to the advances in treatment and diagnostic technologies [2]. Studies have manifested that the occurrence and development of colon carcinoma are related to many factors, like tumor suppressor gene inactivation, proto-oncogene mutation, microsatellite instability, gene overexpression, mismatch repair gene mutation, signal transduction disorder, genome epigenetic modification, and the environment [5–7]. Nonetheless, the specific pathogenesis and molecular mechanisms of colon carcinoma are not fully understood. In-depth study of the pathogenesis of colon carcinoma and identification of potential therapeutic targets have therefore become a hot spot in the medical oncology field.

The noncoding RNAs (ncRNAs) in the transcriptome including microRNAs (miRNAs) are not involved in encoding proteins. Accumulating evidence indicated that ncRNAs could regulate cancer development and progress [8–11]. The studies demonstrated that the aberrant expression and vital roles of miR-339-5p in cancers. For example, miR-339-5p overexpression restrains cell invasion and migration in non-small cell lung cancer (NSCLC). This miRNA is also negatively relevant to lymph node metastasis and TNM stage [12]. Another study reported that miR-339-5p was determined to modulate epithelial-mesenchymal transition through mediating BCL6 expression, thereby affecting cancer cell progression [13]. A few studies have introduced the tumor-suppressing role of miR-339-5p in colorectal cancer [14, 15]. For example, in Chang Zhou's work, miR-339-5p/PRL-1 axis was identified to attenuate the colorectal cancer process [14]. Despite the studies mentioned above, the molecular mechanisms of miR-339-5p are still poorly understood in colon carcinoma.

Collagen Type XI Alpha 1 Chain (COL11A1) is a subtype of fibrillar collagen, which was proved to be related to cancer cell proliferation, migration, and tumorigenesis [16]. COL11A1 is also thought to be a potential prognostic biomarker for breast cancer [16]. Moreover, previous studies revealed that COL11A1 strengthens cancer cell invasion through cooperating with invasion-sensing biomarkers [17]. One study reported that COL11A1 played an imperative role in tumor cell metastasis in breast cancer [18]. COL11A1 is at a high expression level in adenocarcinoma and squamous cell lung cancer compared with that in corresponding normal lung tissue [19], and also is involved in lymphatic metastasis of breast cancer [20]. Nevertheless, far less has been studied on the effects of COL11A1 on the malignant progression of colon carcinoma.

Here, COL11A1 expression was measured in colon carcinoma, and the relationship between COL11A1 and miR-339-5p was researched. This paper aimed to illustrate the influence of the miR-339-5p/COL11A1 axis on colon carcinoma progression. These results provide more theoretical evidence for finding new biomarkers and targeted treatment methods of colon carcinoma.

## 2. Materials and Methods

### 2.1. Bioinformatics Analysis

Expression data of mature miRNAs (normal: 8, tumor: 450) and mRNA (normal: 41, tumor: 473) in The Cancer Genome Atlas-Colon Adenocarcinoma (TCGA-COAD) were acquired by TCGA (https://portal.gdc.cancer.gov/) database. The difference of COL11A1 expression in normal tissue and colon carcinoma tissue was examined by a *t-test* based on the downloaded mRNA expression data with normal tissue as the control. The difference of COL11A1 expression in different tumor clinical stages was detected by the Wilcox test. Differential analysis (|logFC| > 2, padj < 0.01) was done on miRNAs using “edgeR” package to acquire differential miRNAs. Upstream miRNAs of COL11A1 were predicted by starBase (https://starbase.sysu.edu.cn/), mirDIP (https://ophid.utoronto.ca/mirDIP/index.jsp) and miRDB (https://mirdb.org/). The predicted results were overlapped with differentially downregulated miRNAs to select miRNAs having a significant correlation with COL11A1 for research.

### 2.2. Cell Culture

The normal human colon mucosal epithelial cell line NCM460 and the human colon carcinoma cell lines HCT116, Caco205, SW620, and SW480 were bought from the Chinese Academy of Sciences (China). The above cell lines were cultivated with Dulbecco's Modified Eagle Medium (DMEM) containing 10% fetal bovine serum (FBS) and 1% streptomycin/penicillin. All of them were kept at 37°C with 5% CO_2_.

### 2.3. Cell Transfection

SW620 or SW480 cells were incubated in 6-well plates (1 × 10^6^ cells/well) for 24 h. Lipofectamine 2000 reagent (Invitrogen, USA) was employed to transfect si1-COL11A1, si2-COL11A1, oe-COL11A1, miR-mimics or their negative controls (NCs) into colon carcinoma SW620 and SW480 cells.

### 2.4. Real-Time Polymerase Reaction Chain Analysis

Table 1 shows all primer sequences. The RNAiso Plus (TaKaRa Bio Technology, China) was adopted for total RNA separation. 2 *μ*g extracted RNA was transcribed into cDNA by AMV reverse transcriptase (Promega, USA). ABI 7500 real-time detection system (Applied Biosystems by Life Technologies, USA) was utilized to conduct qRT-PCR. 2^−ΔΔCt^ method was employed to measure gene expression between experimental and control groups.

### 2.5. Western Blot Assay

After proteins were collected from cells and separated with 10% sodium dodecyl sulfate polyacrylamide gel electrophoresis, they were mounted onto a polyvinylidene fluoride membrane (Millipore, USA). Afterward, the membrane was incubated with primary rabbit anti-COL11A1 (ab64883, 1 : 500, Abcam, UK) and anti-GAPDH antibodies (ab181602, 1 : 10000, Abcam, UK) at 4°C overnight. After being washed, the membrane was incubated with secondary antibody goat antirabbit IgG (ab6721, Abcam, UK) for 1 h at room temperature. Next, protein bands were detected with enhanced chemiluminescent plus reagents.

### 2.6. Cell Counting Kit (CCK-8) Assay

SW480 and SW620 cells were cultured in 96-well plates with each well 5 × 10^4^ cells. Next, 100 **μ**L CCK-8 solution (Dojindo Molecular Technologies, Japan) was supplemented to each well at 0, 24, 48, 72, and 96 h. Absorbance was assessed at 450 nm with a microplate reader (BioTek, Instruments, USA).

### 2.7. Colony Formation Assay

Cells were incubated with 5% CO_2_ at 37°C for 10 d. Thereafter, colonies (0.3–1.0 mm diameter) were fixed with methyl alcohol, dyed with crystal violet, and counted. Colonies with more than 50 cells were recorded.

### 2.8. Cell Invasion Assay

After transfecting for 48 h, cells were suspended in serum-free DMEM. Thereafter, cells (1 × 10^5^) were seeded in a Transwell chamber (24-well) using inserts with 8 *μ*m pore membranes precoated with Matrigel (BD Biosciences, USA). DMEM containing 10% FBS was placed in the lower chamber. 48 h later, invading cells were fixed with 70% methyl alcohol and dyed with 0.1% crystal violet (Sigma, USA). Finally, cells were counted under a light microscope (400x).

### 2.9. Wound Healing Assay

Firstly, transfected cells were seeded in 6-well plates for culture until 90% confluency. The cell monolayer was scraped by a sterile pipette tip upon fusion. Afterward, cells were washed with a growth medium twice and cultured in serum-free DMEM. Images of the wound area were taken at different time points under a microscope. Wound healing was measured at 0 and 24 h. Statistical data were analyzed according to 6 analyses of each experiment.

### 2.10. Dual-Luciferase Assay

Wild type (WT) COL11A1 3'UTR with binding sites of miR-339-5p or corresponding mutant (MUT) COL11A1 3'UTR was cloned into pGL3 basic vectors (Promega). Afterward, luciferase plasmids were cotransfected into colon carcinoma cells with miR-NC or miR-mimics. Lastly, the Dual-Glo Luciferase Assay System (Promega) was introduced to detect and analyze luciferase activity.

### 2.11. Statistical Analysis

Measurement data of each group were presented and analyzed by mean ± standard error of the mean. The above assays were all repeated for 3 times independently. Average differences between the two groups were analyzed by a *t-test*. Statistical significance was determined when *p* < 0.05. Data were analyzed using GraphPad Prism 6.0 (USA).

## 3. Results

### 3.1. COL11A1 is at Remarkable High Expression Level in Colon Carcinoma

**Figure 1:**
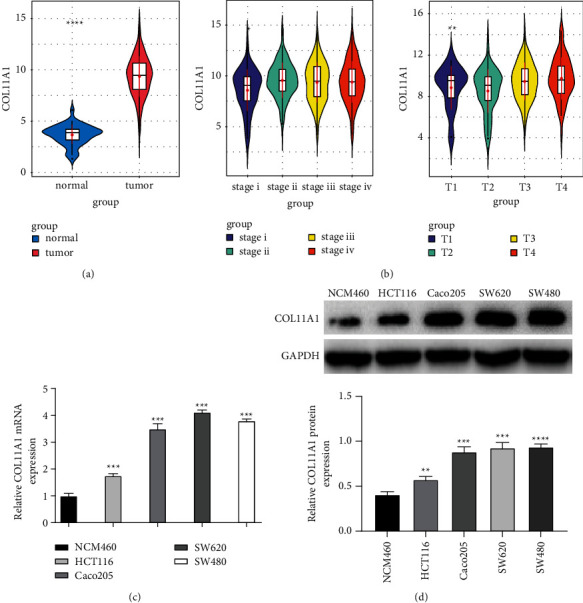
High level of COL11A1 in colon carcinoma. (a) COL11A1 expression differences in TCGA database. (b) COL11A1 expression in different clinical stages and T stages of colon carcinoma. ((c)-(d)) COL11A1 expression was upregulated in colon carcinoma cell lines ^*∗∗∗* ^*p* < 0.001.

As shown in mRNA expression data in the TCGA-COAD dataset, COL11A1 expression was upregulated in colon carcinoma tissue than that in adjacent normal tissue (Figure 1(a)). Further analysis of the patient's clinical information revealed higher COL11A1 expression in Stage IV and T4 stages than in other stages, and the expression elevated as tumor stage increased (Figure 1(b)). Likewise, the COL11A1 expression level in different colon carcinoma cell lines was confirmed through qRT-PCR and western blot, indicating that COL11A1 was remarkably upregulated in colon carcinoma cell lines with normal cell lines as the control (Figures 1(c) and 1(d)). As mentioned above, COL11A1 expressed highly in colon carcinoma cells.

### 3.2. COL11A1 Downregulation Represses Colon Carcinoma Cell Proliferation, Migration, and Invasion

**Figure 2:**
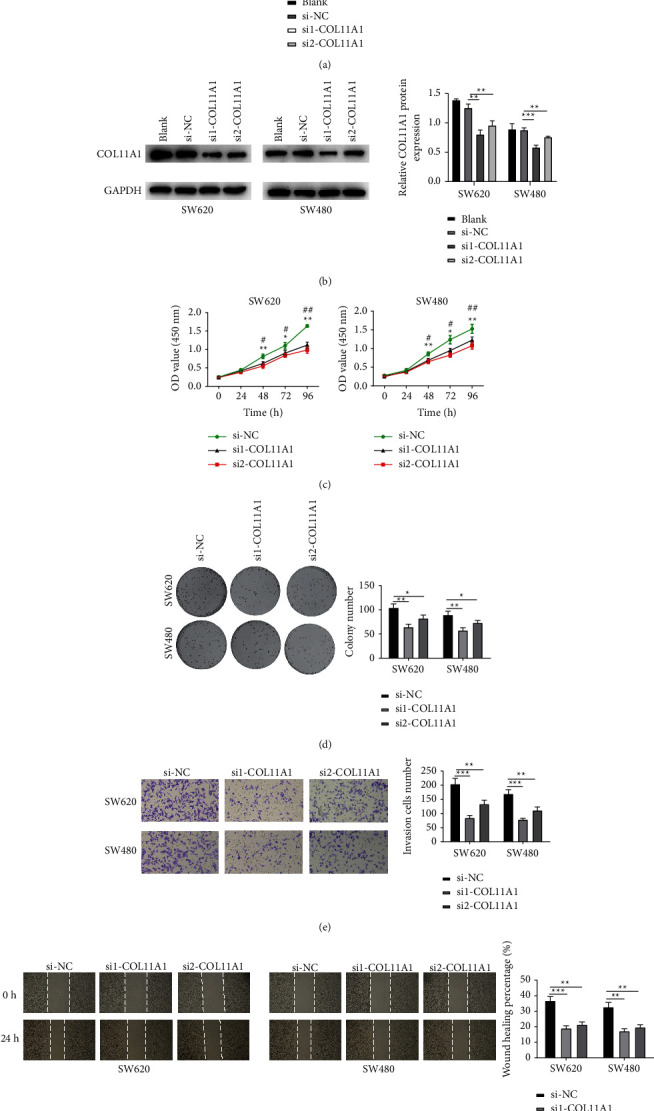
COL11A1 downregulation constrains colon carcinoma cell proliferation, migration, and invasion. (a) COL11A1 mRNA expression in SW620 and SW480 cells in si-NC and si-COL11A1 groups. (b) COL11A1 protein expression in SW620 and SW480 cells. (c) Viability of SW620 and SW480 cells. (d) Colony formation of SW620 and SW480 cells. (e) The invasive ability of SW620 and SW480 cells (100x). (f) The migratory ability of SW620 and SW480 cells (40x). ^*∗*^*p*  < 0.05, ^*∗∗*^*p*  < 0.01, ^*∗∗∗*^*p* < 0.001, #*p*  < 0.05, ^##^*p*  < 0.01, ^###^*p* < 0.001, ^*∗*^ denotes the comparison between si-NC and si1-COL11A1; ^#^ denotes the comparison between si-NC and si2-COL11A1.

We interfered with COL11A1 expression in colon carcinoma cell lines SW620 and SW480. Detection of COL11A1 expression in different groups by qRT-PCR showed that COL11A1 level was remarkably downregulated upon siRNA treatment (Figure 2(a)), indicating a good transfection efficiency. The COL11A1 protein level, as measured by Western blot, was markedly downregulated in cells treated with siRNA (Figure 2(b)). Following this, outcomes of CCK-8 and colony formation methods discovered that COL11A1 inhibition remarkably suppressed the viability and colony formative ability of colon carcinoma cells (Figures 2(c) and 2(d)). Thereafter, the Transwell assay found that cell invasive ability was significantly downregulated after downregulating COL11A1 (Figure 2(e)). Similarly, a wound healing assay discovered that the cell migratory ability was suppressed after downregulating COL11A1 (Figure 2(f)). The above results indicated that silencing COL11A1 restrained colon carcinoma cell malignant behaviors.

### 3.3. MiR-339-5p Binds COL11A1 in Colon Carcinoma

**Figure 3:**
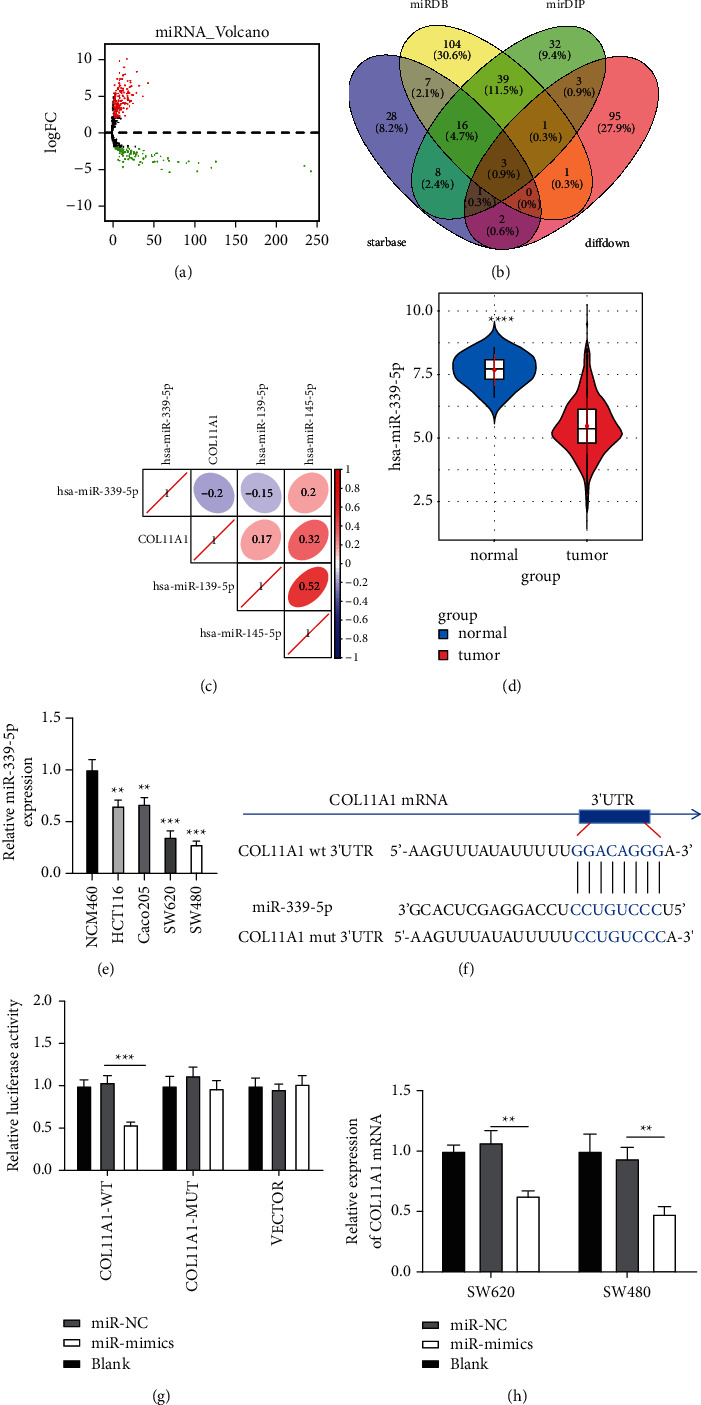
MiR-339-5p binds COL11A1 in colon carcinoma cells. (a) Volcano plot of differential miRNAs from TCGA-COAD. (b) Venn diagram of predicted miRNAs of COL11A1 and differential miRNAs. (c) Pearson correlation analysis of COL11A1 and its predicted upstream miRNAs. (d) Violin plot of miR-339-5p expression from TCGA database. (e) MiR-339-5p expression in NCM460, HCT116, Caco205, SW620, and SW480 cell lines. (f) Bioinformatics predicted binding sites of miR-339-5p and COL11A1. (g) Relative luciferase activity of different transfection group. (h) COL11A1 mRNA expression level in colon carcinoma SW620 and SW480 after overexpressing miR-339-5p. ^*∗*^*p*  < 0.05, ^*∗∗*^*p*  < 0.01, ^*∗∗∗*^*p* < 0.001.

Afterward, the upstream regulatory miRNAs of COL11A1 were predicted by databases. Differential analysis was undertaken on TCGA data with the EdgeR package and 299 differential miRNAs were obtained (upregulated: 193, downregulated: 106) (Figure 3(a)). The predicted results were overlapped with 106 differentially downregulated miRNAs. 3 miRNAs that shared binding sites with COL11A1 were obtained (miR-139-5p, miR-145-5p, and miR-339-5p) (Figure 3(b)). Among them, miR-339-5p manifested the strongest correlation coefficient with COL11A1 (Figure 3(c)). MiR-339-5p was markedly lowly expressed in colon carcinoma tissue as presented in TCGA (Figure 3(d)). qRT-PCR discovered that miR-339-5p expressed markedly low in colon carcinoma cells HCT116, Caco205, SW620, and SW480 (Figure 3(e)). The binding sites of miR-339-5p and COL11A1 were predicted (Figure 3(f)). Hence, it was speculated that miR-339-5p could bind COL11A1. To that end, a dual-luciferase assay proved that miR-339-5p overexpression repressed luciferase activity of WT COL11A1 mRNA 3'-UTR, while no effect was found on that of MUT COL11A1 mRNA 3'-UTR (Figure 3(g)). Besides, qRT-PCR assay manifested that miR-339-5p overexpression declined COL11A1 expression in colon carcinoma cells (Figure 3(h)). Based on the experimental data, COL11A1 was confirmed as a downstream target of miR-339-5p in colon carcinoma.

### 3.4. MiR-339-5p Represses Colon Carcinoma Cell Proliferation, Migration, And Invasion via Modulating COL11A1

**Figure 4:**
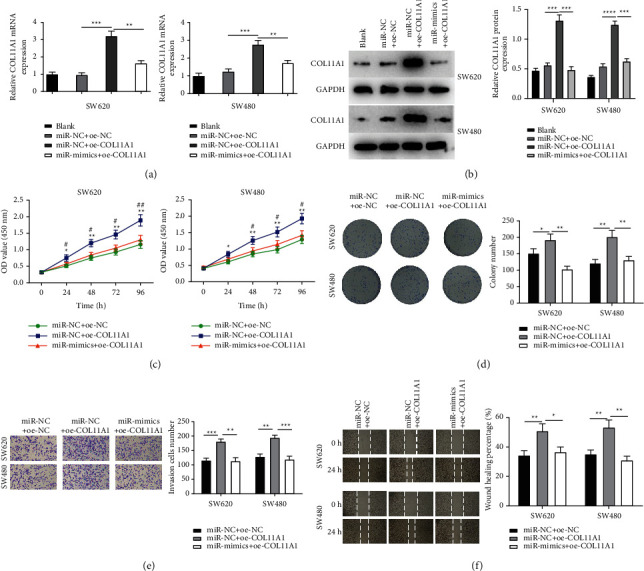
MiR-339-5p suppresses colon carcinoma cell progression through COL11A1 downregulation. (a-b) COL11A1 mRNA and protein levels in colon carcinoma cells SW620 and SW480 in different treatment groups. (c) The viability of SW620 and SW480 cells. (d) The colony formation of SW620 and SW480 cells. (e) The invasive ability of SW620 and SW480 cells (100x). (f) The migratory ability of SW620 and SW480 cells (40x). ^*∗*^*p*  < 0.05, ^*∗∗*^*p*  < 0.01, ^*∗∗∗* ^*p* < 0.001, ^#^*p*  < 0.05, ^##^*p*  < 0.01, ^###^*p* < 0.001, ^*∗*^denotes the comparison between miR-NC + oe-NC and miR-NC + oe-COL11A1; ^#^ denotes the comparison between miR-NC + oe-COL11A1 and miR-mimics + oe-COL11A1.

To explore whether miR-339-5p participates in regulating colon carcinoma cells via targeting COL11A1, we constructed miR-NC + oe-NC, miR-NC + oe-COL11A1, and miR-mimics + oe-COL11A1 groups. As measured by qRT-PCR and Western blot assays, COL11A1 levels were markedly upregulated in the oe-COL11A1 group. However, COL11A1 expression was recovered in miR-mimics + oe-COL11A1 (Figures 4(a) and 4(b)), illustrating that COL11A1 level could be downregulated by miR-339-5p. CCK-8 assay showed that COL11A1 overexpression remarkably promoted viability of colon carcinoma cells, yet the promotion was repressed by simultaneously overexpressing miR-339-5p (Figure 4(c)). Colony formation assay also displayed that COL11A1 overexpression remarkably elevated colony formative ability of cancer cells, while simultaneously overexpressing COL11A1 as well as miR-339-5p greatly reduced the ability (Figure 4(d)). Afterward, it was discovered by Transwell and wound healing assays that the invasive and migratory functions of cancer cells were remarkably elevated after overexpressing COL11A1. However, the invasion and migration were reduced in the miR-mimics + oe-COL11A1 group compared with those in the miR-NC + oe-COL11A1 (Figures 4(e) and 4(f)). Together, miR-339-5p repressed tumor cell functions through COL11A1 downregulation.

## 4. Discussion

Several previous works pointed out high COL11A1 expression in colon carcinoma tissues. For instance, COL11A1 expression was markedly high in colorectal cancer tissue [21]. Similarly, Fisher's team identified the high COL11A1 expression in familial adenomatosis, which may be associated with activation of ACP/beta-catenin pathway [22]. One study applied bioinformatics method to research the prognostic value of COL11A1 in colorectal carcinoma [23]. Nonetheless, the above works focused much more on COL11A1 expression status in colorectal cancer. Considering COL11A1 molecular mechanisms in colon cancer, we designed the experiments to determine the effects of COL11A1s on cancer-related cellular behaviors, which is a novelty of this study compared with the previous reports.

As mentioned in the introduction section, miR-339-5p shows a tumor-suppressing role among various cancers via diverse pathways. Also, consistent with the previous studies, this paper proved the tumor-inhibiting effects of miR-339-5p to some extent. However, no study has reported the effect of COL11A1 and the way the miR-339-5p/COL11A1 axis worked in colon carcinoma. This paper found that forced miR-339-5p expression suppressed COL11A1-induced tumor-promoting effect. A dual-luciferase assay identified the binding of miR-339-5p and COL11A1. Therefore, miR-339-5p restrained colon carcinoma cell behaviors by targeting COL11A1.

To sum up, this work demonstrated the influence of miR-339-5p on inhibiting colon carcinoma cell progression. MiR-339-5p suppressed cancer via downregulating COL11A1 expression, and COL11A1 promoted colon carcinoma progression. The miR-339-5p/COL11A1 regulatory axis could participate in colon carcinoma progression and may be used as a potential therapeutic indicator. However, several limitations remain in the current study. First, we failed to present the miR-339-5p/COL11A1 regulatory axis *in vivo*, which would be robust proof for this mechanism. Second, we did not process in-depth research to identify COL11A1 downstream regulations in colon cancer. Considering the limitations, we are planning to carry on an *in-vivo* animal experiment.

## Figures and Tables

**Table 1:** qRT-PCR primer sequences.

Gene		Sequence
miR-339-5p	Forward primer	5′-CGCTCTCCCTGTCCTCCA-3′
	Reverse primer	5′-GCACACGTGAGCTCCTGG-3′

U6	Forward primer	5′-CTCGCTTCGGCAGCACA-3′
	Reverse primer	5′-AACGCTTCACGAATTTGCGT-3′

COL11A1	Forward primer	5′-ACCTGACCTGCCGTCTAGAA-3′
	Reverse primer	5′- TCCACCACCCTGTTGCTGTA-3′

GAPDH	Forward primer	5′-GTGACGATGAACTTCGACTT-3′
	Reverse primer	5′-GGCGTTTGGAGTGGTAGAAATC-3′

## Data Availability

The data used to support the findings of this study are included within the article.
